# Influence of *Citrobacter freundii* on *NINJ2* Expression and Oxaliplatin Resistance in Colorectal Cancer

**DOI:** 10.1002/cam4.70940

**Published:** 2025-06-26

**Authors:** Reio Ueta, Hiroo Imai, Ken Saijo, Yoshifumi Kawamura, Shuto Kodera, Chikashi Ishioka

**Affiliations:** ^1^ Department of Clinical Oncology Tohoku University Graduate School of Medicine Sendai Japan

**Keywords:** bacterial culture filtrate, *Citrobacter freundii*, colon cancer, gut microbiome, oxaliplatin

## Abstract

**Background:**

Oxaliplatin, a third‐generation platinum‐based chemotherapeutic agent, is widely used in the treatment of colorectal cancer (CRC). However, some patients do not respond effectively to oxaliplatin, and intrinsic resistance to the drug poses a significant challenge. Recent studies have revealed an association between the gut microbiome and the progression of CRC. We hypothesized that *Citrobacter freundii*, a component of the gut microbiome, contributes to oxaliplatin resistance by regulating specific gene expression in CRC cells.

**Methods:**

A bacterial culture filtrate from *Citrobacter freundii* was employed in the experiments. The CRC cell line RKO, following exposure to this filtrate, was analyzed using high‐throughput RNA sequencing. Candidate genes were identified through MTT assays, siRNA knockdown, and overexpression experiments. Apoptosis and reactive oxygen species (ROS) assays were performed to investigate the underlying mechanisms. Finally, a xenograft mouse model was used to evaluate oxaliplatin resistance in vivo.

**Results:**

Exposure to bacterial culture filtrate from *Citrobacter freundii* induced oxaliplatin resistance in RKO cells with downregulation of the *NINJ2* gene as a possible molecular mechanism. Reduced*NINJ2* gene expression suppressed oxaliplatin‐induced apoptosis and ROS generation. A tendency toward reduced oxaliplatin efficacy was observed in vivo when *NINJ2* gene expression was suppressed.

**Conclusion:**

This study demonstrates that *Citrobacter freundii* promotes oxaliplatin resistance in CRC through downregulation of *NINJ2* gene. *NINJ2* gene may serve as a predictive biomarker and therapeutic target to overcome oxaliplatin resistance in CRC.

## Graphical abstract

Oxaliplatin resistance was found to be induced in colon cancer cells by bacterial filtrates from *Citrobacter freundii* . This resistance was associated with downregulation of the NINJ2 gene, which was found to suppress apoptosis and reactive oxygen species.

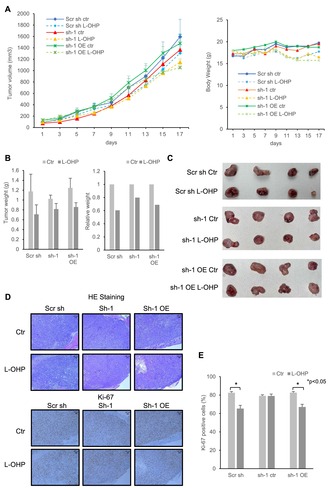

**Abbreviations**
DCFH‐DA: 2′,7′‐Dichlorodihydrofluorescein diacetate
DNA: deoxyribonucleic acid
HE: hematoxylin–eosin
miRNA: microRNA
mRNA: messenger RNA
NC: negative control
OE: overexpression
RNA: ribonucleic acid
shRNA: short hairpin RNA
siRNA: small interfering RNA

## 1 Introduction

Colorectal cancer (CRC) is one of the most prevalent malignancies worldwide [1]. Approximately 20% of CRC patients are already in an advanced, unresectable stage with distant metastases before treatment [2], and systemic chemotherapy is commonly used for these cases. With recent advancements in anticancer therapy, the median survival time for unresectable CRC with anticancer therapy has exceeded 30 months [3, 4]. Oxaliplatin, a cytotoxic anticancer drug, is a platinum‐based drug that elicits anticancer effects by binding to DNA and creating cross‐links [5]. Oxaliplatin is considered a cornerstone in the treatment of advanced CRC [6]. When utilized in advanced CRC, oxaliplatin‐based chemotherapy displays beneficial therapeutic effects in approximately 50% of patients [3, 7, 8]. However, biomarkers predicting oxaliplatin's therapeutic efficacy or the mechanisms behind its resistance remain elusive, making research on these topics paramount.

The gut microbiome plays a pivotal role in regulating immunity, metabolic activity, and nutrient absorption within the human body [9, 10, 11]. Emerging research indicates that the gut microbiome influences cancer progression and therapeutic outcomes, especially in CRC [12, 13]. Enterotoxigenic *Bacteroides fragilis* , a pathogenic strain of *Bacteroides fragilis* , is associated with colorectal tumorigenesis [14]. In addition, pks‐positive *Escherichia coli* , which produces the genotoxin colibactin, causes colorectal cancer by inducing DNA double‐strand breaks [15]. Bacterial species such as *Porphyromonas asaccharolytica* and *Porphyromonas gingivalis* are identified in increased amounts in the intestines of patients with CRC. It is reported that butyric acid, produced by these bacteria, can modify the tumor microenvironment, thereby promoting CRC progression [16]. Moreover, comprehensive DNA and RNA analyses have revealed elevated levels of *Fusobacterium nucleatum* in CRC tissues compared to normal tissues [17]. It is documented that FadA, a cell adhesion protein found on *Fusobacterium nucleatum* , binds to E‐cadherin (a cellular adhesion factor), subsequently activating β‐catenin signaling to promote cell proliferation [18].

Connections between the gut microbiome and the effectiveness of anticancer drugs are becoming evident. For instance, *Fusobacterium nucleatum* triggers an innate immune response via TLR4 (toll‐like receptor 4) and MYD88 (myeloid differentiation factor 88), subsequently activating the autophagy pathway. This inhibits apoptosis, leading to resistance against anticancer drugs [19]. Another study suggests that *Escherichia coli* impacts the efficacy of 5‐fluorouracil treatment by altering VitB6, VitB9, and RNA‐mediated pathways [20].

In 2017, Geller and colleagues isolated bacteria from excised pancreatic cancer tissues and discerned that such bacteria induced resistance to gemcitabine [21]. In this study, they identified *Citrobacter freundii* as a factor contributing to oxaliplatin resistance. *Citrobacter freundii* is a gram‐negative anaerobic bacterium belonging to the Enterobacteriaceae family [22]. It constitutes the gut microbiome of humans and is also detected in the intestinal tract of animals [23, 24]. Although the proportion of *Citrobacter freundii* within the total gut microbiome is relatively low, the proportion may increase due to opportunistic infections and disturbances of the gut microbiome [25]. While they demonstrated that the bacterial enzyme cytidine deaminase degrades gemcitabine, the mechanism by which *Citrobacter freundii* induces resistance to oxaliplatin remains unclear [21]. Geller et al. focused on the role of the intratumoral microbiome in chemoresistance. However, they did not examine the relationship between the intratumoral microbiome and the gut microbiome. Several studies have reported differences in bacterial distribution between tumor tissues and non‐tumor tissue. In colon tumors, *Fusobacterium nucleatum* was abundant in adenoma, but not in adjacent normal tissues [26]. In gastric cancer, the richness and diversity of microorganisms increased in tumor tissues more than in the intestinal tract [27]. Despite these distinctions, emerging evidence suggests a potential connection between the intratumoral and gut microbiome. An in vivo study demonstrated that *Bifidobacterium*, when administered intravenously, can travel through the bloodstream and accumulate in tumors [28]. In pancreatic cancer, certain components of the gut microbiome are also present within tumors [29], and transplantation of the fecal microbiome can alter not only the gut but also the intratumoral microbiome [30]. Furthermore, metabolites derived from the gut microbiome can reach pancreatic tumors via the bloodstream and influence chemosensitivity [31]. In colorectal cancer, Yu et al. reported that *Fusobacterium nucleatum* translocated from the intestinal tract into tumor tissue and induces chemoresistance [19]. From these reports, the intratumoral microbiome and the gut microbiome seem to be closely related, but the direct or indirect mechanisms underlying the relationship between the two remain unclear.

Extracellular vesicles (EVs) are membrane‐bound vesicles, ranging from 50 to 5000 nm in diameter, secreted by nearly all cells. They encompass miRNA, other RNAs, DNA, proteins, and metabolites [32]. Such vesicles are acknowledged for influencing tumor cells [33]. For instance, small RNAs transported by these vesicles have been linked to cancer metastasis [34]. EVs also promote angiogenesis and increase vascular permeability, paving the way for tumor expansion and metastasis [35, 36]. Notably, almost all bacteria, especially Gram‐negative species, are recognized to produce EVs [37]. From these findings, it can be hypothesized that *Citrobacter freundii* , a gram‐negative bacillus, generates EVs that impact tumors. Centrifugation and sterile filtration are commonly adopted to collect EVs [38, 39], and various reports have delved into the sensitivity of multiple anticancer drugs across different cancer types using bacterial culture filtrates [40]. Hence, our objective was to decode the mechanism behind oxaliplatin resistance using bacterial culture filtrates derived from *Citrobacter freundii* . This research was initiated to elucidate the mechanisms by which the gut microbiome, specifically *Citrobacter freundii* , contributes to oxaliplatin resistance, aiming to foster novel therapeutic strategies for CRC treatment.

## 2 Methods

### 2.1 Reagents

Oxaliplatin, carboplatin, cisplatin, and 5‐fluorouracil were acquired from Fujifilm Wako Pure Chemicals Corporation (Osaka, Japan). For in vitro assays, reagents were dissolved in water, while for in vivo assays, they were dissolved in a 5% glucose solution.

### 2.2 Cell Lines

The human CRC cell lines RKO, DLD1, HT29, and HCT‐116 were used in this study. RKO was acquired from the American Type Culture Collection (Manassas, VA, USA). HCT‐116 was sourced from the RIKEN Bio Resource Research Center (Ibaraki, Japan). DLD1 and HT29 cells were kindly provided by Dr. John M. Mariadason (Ludwig Institute for Cancer Research, Melbourne). All cell lines were cultured in Roswell Park Memorial Institute (RPMI) 1640 medium supplemented with 10% fetal bovine serum. Unless otherwise specified, all in vitro experiments in this study were repeated three times.

### 2.3 Bacterial Culture Filtrate

*Citrobacter freundii* and *Pseudomonas alcaliphila* were procured from KAC (Kyoto, Japan). The bacteria were introduced into 30 mL of RPMI medium and incubated on a shaker at 37°C for 24 h. Following incubation, the medium underwent centrifugation at 3000 rpm for 15 min. Subsequently, the supernatant was passed through a 0.2 μm filter (Advantech Toyo, Tokyo, Japan). The pH of the supernatant was adjusted using 5 M NaOH. This bacterial culture filtrate was freshly prepared for each experiment to account for pH changes and proteolysis due to freezing.

### 2.4 Cell Viability Assays

Cell viability was determined using the MTT assay. Cells were seeded in 24‐well plates at a density of 3.0 × 10^4^ cells/well and incubated for 24 h. The medium was then replaced with a drug‐containing medium specific to each experimental condition. For the control group, the medium was replaced with medium containing the same volume of water used to dissolve the drugs in the treatment groups. After a further 24‐h incubation, the cells were treated with the appropriate drugs and cultured for an additional 48 h. Following this, the cells were incubated with the Cell Counting Kit‐8 (Dojindo Laboratories, Kumamoto, Japan) for 2 h. Absorbance was then measured at 450 nm utilizing the SpectraMax M2e (Molecular Devices, CA, USA).

### 2.5 RNA Sequencing

RKO cells were seeded in 24‐well plates at a density of 3.0 × 10^4^ cells/well and incubated for 24 h. After this incubation period, the medium was replaced with RPMI medium for the control group and with a 1:1 mixture of RPMI medium and bacterial culture filtrate for the bacterial culture filtrate group. Subsequent incubation continued for an additional 48 h. RNA from cells in each group was extracted using the RNeasy Micro Kit (Qiagen, Hilden, Germany), with three samples taken from each group. RNA sequencing (RNA‐seq) was performed by DNA Chip Research Inc. (Tokyo, Japan). Gene expression ratios derived from RNA‐seq were presented as log_2_ fold‐change on the x‐axis, while the *p*‐value was transformed to log worth and plotted on the y‐axis. Genes with a log worth exceeding 1.3 and a fold‐change below‐2 at the 0.05 significance level were identified as significantly downregulated genes. The gene expression data were then analyzed utilizing JMP Pro ver. 17.0 (SAS Institute, NC, USA).

### 2.6 Western Blot Analysis

Cells were seeded in a 6‐well plate at a density of 2.0 × 10^5^ cells/well and allowed to incubate for 24 h. Post‐incubation, the cells were treated with oxaliplatin and incubated for an additional 48 h. Following this incubation, proteins were extracted from the cells using the radioimmunoprecipitation assay (RIPA) buffer. These proteins were then separated by polyacrylamide gel electrophoresis and subsequently transferred onto a polyvinylidene difluoride membrane (PVDF membrane, Merck Millipore Ltd., MA, USA). The PVDF membrane was then blocked with Intercept Blocking Buffer (Licor Inc., NE, USA) for 1 h at room temperature. After the blocking step, the membrane was incubated with the primary antibody solution overnight at 4°C. The membrane was then washed three times for 5 min with Tris Buffered Saline with Tween 20 (TBS‐T). It was subsequently incubated with the Alexa Fluor 680 secondary antibody (Thermo Fisher Scientific Inc.) at room temperature for 1 h. After another set of 3, 5‐min washes with TBS‐T, the signal was detected using the Odyssey Infrared Imaging System (Licor Inc). Antibodies against Poly Adenosine Diphosphate‐ribose Polymerase (PARP) and p38 Mitogen‐Activated Protein Kinase (MAPK), Phospho‐p38 MAPK (Thr345/Ser346) were sourced from Cell Signaling (MA, USA). Additionally, antibodies against α‐tubulin and β‐actin were purchased from Sigma Aldrich (MO, USA).

### 2.7 NINJ2 Knockdown and Overexpression

The siRNAs employed in this research were Silencer Select Pre‐Designed siRNAs (*NINJ2* ID: s9559, s9560, s224118), (*C1orf53* ID: s52248), (*HIST1H2AC* ID: 13260), (*HIST1H2AD* ID: s230194), (*HIST1H2BD* ID: 224292), (*HIST1H3E* ID: s194909), (*HIST1H4H* ID: 224292), (*RAD9B* ID: s44687), and (*TMEM88* ID: 149446), all sourced from Thermo Fisher Scientific Inc. Control cells (NC si) were transfected with AccuTarget Negative Control siRNA (Bioneer, Seoul, Korea). Cells were seeded in 24‐well plates at a density of 1.0 × 10^4^ cells/well and cultured for 24 h. Transfection was performed using Opti‐MEM transfection reagent (Thermo Fisher Scientific Inc.) in combination with Lipofectamine RNAiMAX Transfection Reagent (Thermo Fisher Scientific Inc.), administering 1.2 μL per well, with the siRNA adjusted to achieve a final concentration of 6 pM.

Scramble shRNA (Control shRNA Lentiviral Particles‐A sc‐108080) and *NINJ2* shRNA (*Ninjurin‐2* shRNA(h) Lentiviral Particles SC‐75917‐V) were procured from Santa Cruz (CA, USA). The lentiviral vector utilized for *NINJ2* overexpression in this study, pLV[Exp]‐EGFP:T2A:Bsd‐EF1A>h*NINJ2*[NM_016533.6] (ID: VB900138‐5018ejv), was constructed and packaged by Vector Builder Inc. (Chicago, IL, USA).

Cells were seeded in 12‐well plates at a density of 4.0 × 10^4^ cells/well and were cultured for an additional 24 h. Thereafter, the medium was substituted with a combination of polybrene (Nacalai Tesque, Kyoto, Japan) and each set of lentiviral particles, incubating the cells for 4 h. Scrambled shRNA and *NINJ2* shRNA were transduced into RKO cells, followed by the addition of puromycin (InvivoGen, San Diego, USA) to select stable cell lines. The RKO cells transfected with *NINJ2* shRNA were further infected with lentiviral particles overexpressing the *NINJ2* gene. Blasticidin (Fuji Film Wako Pure Chemicals) was used for the selection of stable cell lines.

### 2.8 Quantitative Real‐Time PCR Assay (qPCR)

Cells were seeded in a 24‐well plate at a density of 3.0 × 10^4^ cells/well and cultured for 24 h. Total RNA was extracted from the cells using the RNeasy Micro Kit (Qiagen). Reverse transcription reactions were performed using the iScript Advanced cDNA Synthesis Kit for RT‐qPCR (Bio‐Rad Laboratories Inc., CA, USA) at 42°C for 30 min to produce reverse transcription products. Primers for *NINJ2* (forward: 5′‐CATCCTCTCACTACTACACCACC‐3′, reverse: 5′‐CTGGTTGAGTCGCCACTGCTTT‐3′) and *GAPDH* (forward: 5′‐TCAGCGGCATCTTCTTTTGC‐3′, reverse: 5′‐TTAAAAGCAGCCCTGGTGAC‐3′) were designed and synthesized by Hokkaido System Science (Hokkaido, Japan). The final primer concentration was 0.4 μM. TB Green Premix EX TaqII (Bio‐Rad Laboratories Inc.) was used as the qPCR reagent. The qPCR assay was performed on the CFX96 Touch Deep Well Real‐Time PCR Analysis System (Bio‐Rad Laboratories Inc.). The ΔΔCt method was used to quantify relative mRNA expression, utilizing *GAPDH* mRNA as the internal control.

### 2.9 Annexin V Assay

Cells were seeded in 6‐well plates at a density of 1.0 × 10^5^ cells/well. After 24 h of incubation, oxaliplatin was added at a concentration of 5 μM and the cells were incubated at 37°C for an additional 48 h. Thereafter, the cells were harvested with trypsin, rinsed with phosphate‐buffered saline (PBS), and stained utilizing the Annexin V‐633 Apoptosis Detection Kit (Nacalai Tesque). Apoptotic cells were quantified using CytoFLEX (Beckman Coulter Inc).

### 2.10 Reactive Oxygen Species (ROS) Assay

Cells were seeded in 6‐well plates at a density of 1.0 × 10^5^ cells/well. After 24 h of incubation, oxaliplatin was added at a concentration of 5 μM, and the cells were incubated at 37°C for an additional 48 h. Post incubation, cells were harvested using trypsin, rinsed with PBS, and stained using the Highly Sensitive DCFH‐DA ROS Assay Kit (Dojindo Laboratories). Following a 30‐min incubation at 37°C, measurements were performed using CytoFLEX (Beckman Coulter Inc.).

### 2.11 Xenograft Mouse Model

Female nude mice (BALB/c‐nu, 4 weeks old) were procured from The Jackson Laboratory Japan (Yokohama, Japan). Cells were harvested using trypsin and combined in a 1:1 ratio with RPMI medium and Corning Matrigel basement membrane matrix (Corning, NY, USA) to achieve a density of 1.0 × 10^7^ cells/mL. The mice were divided into three groups: Scrambled shRNA (Scr sh), *NINJ2* shRNA (sh‐1), and *NINJ2* overexpression (sh‐1 OE), with each group comprising eight mice. Then, 0.1 mL of the cell suspension from each group was subcutaneously injected into the right flank of the respective mice.

Once the tumors became measurable, four mice from every group were randomly designated to the oxaliplatin treatment group, while the remaining four were assigned to the control group. Oxaliplatin was dissolved in a 5% glucose solution and administered intraperitoneally at a dose of 10 mg/kg once weekly for three consecutive weeks. The control groups received a 5% glucose solution. Tumor dimensions and weight were monitored every 2 days from the initiation of the treatment. The tumor volume was estimated using the formula: tumor volume (mm^3^) = 0.5 × length (mm) × width (mm) × width (mm).

### 2.12 Immunohistochemistry

Tumors excised from the xenograft mouse model were fixed in 10% formalin. Immunohistochemical staining, utilizing both HE staining and anti‐Ki‐67 antibody (Cell Signaling), was executed on formalin‐fixed, paraffin‐embedded tissue sections. Under an optical microscope, five regions exhibiting the highest tumor density in each group were chosen at 400× magnification. Subsequently, a minimum of 500 cells from these areas were tallied. The Ki‐67 labeling index was determined as the ratio of Ki‐67 positive cells to total cells.

### 2.13 Statistical Analysis

The Student's *t*‐test was employed to test for significant differences, with a *p*‐value < 0.05 deemed statistically significant. Statistical analyses were performed using JMP Pro ver. 17.0 (SAS Institute) or Microsoft Excel for Microsoft 365 MSO (Microsoft, WA, USA).

## 3 Results

### 3.1 Alteration of Anticancer Drug Resistance by the Addition of Bacterial Culture Filtrate

**FIGURE 1:**
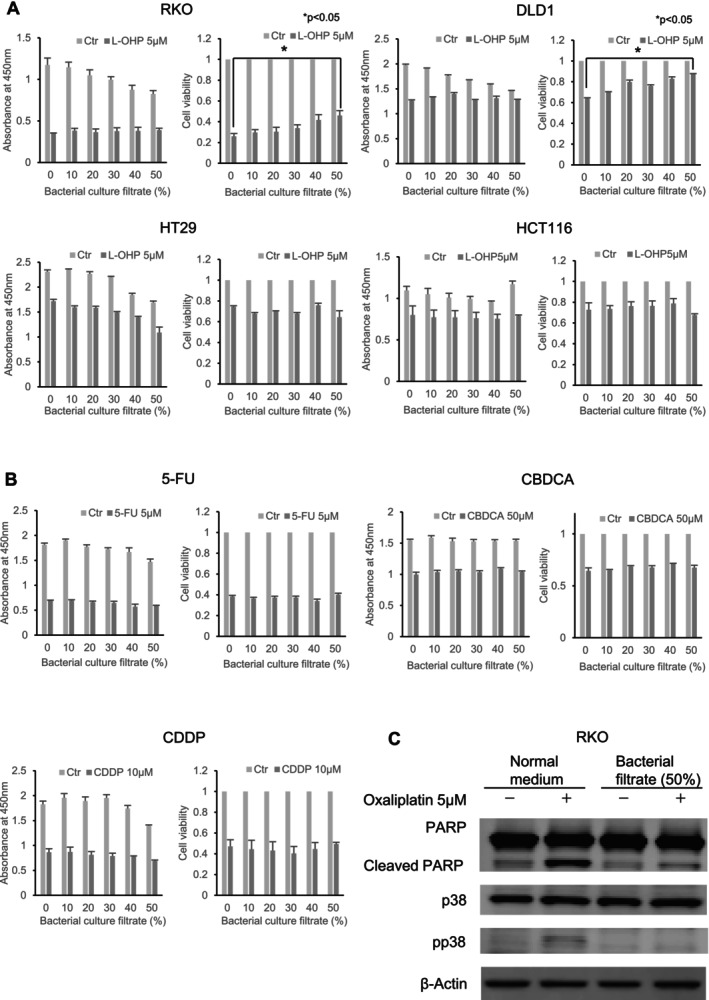
Assessment of oxaliplatin sensitivity influenced by bacterial culture filtrate in colorectal cancer cell lines. (A) RPMI medium and bacterial culture filtrate were combined at specified concentrations (0%, 10%, 20%, 30%, 40%, and 50%). Cells were then incubated and treated with oxaliplatin (L‐OHP) at 5 μM concentration following a 24‐h period. An MTT assay was carried out 48 h post‐oxaliplatin treatment. The resulting absorbance and cell viability are represented on the vertical axis, with cell viability displayed relative to its level at each bacterial culture filtrate concentration *p < 0.05. (B) RPMI medium mixed with bacterial culture filtrate at concentrations (0%, 10%, 20%, 30%, 40%, and 50%) was used to incubate RKO cells for 24 h. Subsequently, the cells were treated with 5‐fluorouracil (5‐FU), carboplatin (CBDCA), and cisplatin (CDDP) at a final concentration of 5 μM, 50 μM, and 10 μM, respectively. MTT assays were then conducted 48 h post‐treatment. Absorbance and cell viability results are plotted on the vertical axis, with cell viability indicated relative to its respective bacterial culture filtrate concentration. (C) Twenty‐four hours after seeding RKO cells, the medium was switched to a mixture of RPMI medium and 50% bacterial culture filtrate. After an additional 24‐h incubation, the cells were treated with oxaliplatin at a final concentration of 5 μM. Cells were harvested 48 h post‐oxaliplatin treatment. Protein extraction was performed on the harvested cells followed by Western blot analysis.

To determine if the sensitivity to oxaliplatin in colon cancer cell lines RKO and DLD‐1 is influenced by the addition of bacterial culture filtrate, MTT assays were conducted. The cell viability of RKO and DLD‐1 was assessed upon the addition of bacterial culture filtrate at various concentrations. Notably, both RKO and DLD‐1 displayed resistance to oxaliplatin in a bacterial culture filtrate concentration‐dependent manner (Figure 1A). In RKO cells, the mean cell viability was 25.9% ± 2.3% (standard deviation, SD) in the medium containing 0% bacterial culture filtrate, whereas in the medium containing 50% bacterial culture filtrate, the mean cell viability increased to 46.1% ± 4.5%. In DLD1 cells, the mean cell viability was 63.8% ± 1.0% in the medium containing 0% bacterial culture filtrate, compared to 87.0% ± 0.5% in the medium containing 50% bacterial culture filtrate. When cell viability was compared in media containing 0% and 50% bacterial culture filtrate, a significant increase in viability was observed in the 50% filtrate media compared to the 0% filtrate media (RKO, *p* = 0.032; DLD‐1, *p* < 0.001). These observations indicate that the antitumor efficacy of oxaliplatin diminishes with rising concentrations of bacterial culture filtrate. Conversely, when oxaliplatin was administered to colon cancer cell lines HT29 and HCT116, no enhancement in cell viability was noted with increasing bacterial culture filtrate concentrations. This suggests that the decline in oxaliplatin's antitumor efficacy upon the addition of bacterial culture filtrate might be specific to certain cell lines.

Further MTT assays were conducted to ascertain if the bacterial culture filtrate‐induced resistance to anticancer drugs was specific to oxaliplatin. Cell viability was assessed by introducing 5FU, a key drug for CRC, and the platinum drugs cisplatin and carboplatin to RKO cells (Figure 1B). Results revealed that the inclusion of bacterial culture filtrate did not confer resistance to these anticancer drugs in RKO cells, suggesting that resistance induced by the bacterial culture filtrate in colon cancer cells might be peculiar to oxaliplatin.

Geller et al. showed that *Citrobacter freundii* was resistant to oxaliplatin, whereas *Pseudomonas alcaliphila* was not [21]. To investigate whether bacterial species other than *Citrobacter freundii* might show oxaliplatin resistance, an MTT assay using the culture filtrate of *Pseudomonas alcaliphila* was performed (Figure S1). The results demonstrated that *Pseudomonas alcaliphila* did not show resistance to oxaliplatin, supporting the findings in the study that oxaliplatin resistance has species specificity.

To validate the influence of bacterial culture filtrate on protein expression in RKO cells, Western blot analysis was performed (Figure 1C). In standard RPMI medium (Normal medium), oxaliplatin treatment augmented the expression of apoptosis‐related proteins, cleaved PARP, and pp38. In contrast, in the presence of bacterial culture filtrate (bacterial filtrate 50%), this upregulation of cleaved PARP and pp38 was curtailed. This outcome implies that introducing bacterial culture filtrate to the medium may hinder oxaliplatin‐induced apoptosis.

### 3.2 Validation of High‐Throughput Gene Expression Analysis by Adding Bacterial Culture Filtrate Using RNA Sequencing

**FIGURE 2:**
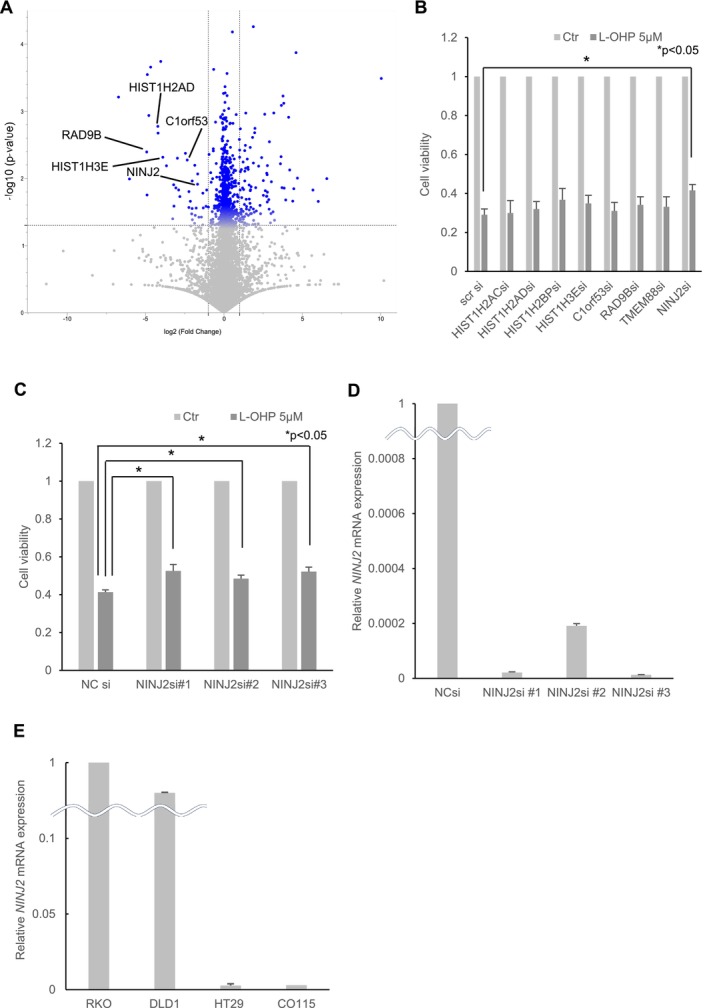
Comprehensive gene analysis associated with oxaliplatin resistance in colon cancer cells. (A) A volcano plot illustrates genes with altered expression levels in RKO cells treated with 50% bacterial culture filtrate compared to those in the RPMI group. The X‐axis represents log_2_ fold‐change, while the Y‐axis indicates –log_10_(*p*‐value). Genes with a fold‐change exceeding 2 and a log worth surpassing 1.3 at a significance level of 0.05 were identified as candidate genes. Pseudogenes are included in the plot. (B) The MTT assay was conducted using siRNAs for the eight selected genes. Oxaliplatin (L‐OHP) was introduced 24‐h post‐transfection at a final concentration of 5 μM. Forty‐eight hours after oxaliplatin exposure, cell viabilities were assessed via the MTT assay. The vertical axis depicts the ratio of (cell viability following oxaliplatin treatment) to (cell viability after saline treatment) *p < 0.05. (C) Cell viability for siRNA‐transfected cells was determined using the MTT assay, with samples from NC siRNA, *NINJ2* si#1, *NINJ2* si#2, and *NINJ2* si#3, respectively. The vertical axis shows the ratio of (cell viability post‐oxaliplatin treatment) to (cell viability post‐saline treatment) *p < 0.05. (D) RKO cells were transfected with NC siRNA, *NINJ2* si#1, *NINJ2* si#2, and *NINJ2* si#3, respectively. Following transfection, RNA was extracted from each set of cells. To evaluate mRNA expression levels, qPCR was then employed. The vertical axis represents the mRNA expression level in cells transfected with *NINJ2* si#1, *NINJ2* si#2, or *NINJ2* si#3 relative to the levels observed in NC siRNA‐transfected cells. (E) RNA was extracted from RKO, DLD1, HT29, and CO115 cells. The vertical axis represents the mRNA expression level in each cell line relative to that in RKO.

High‐throughput gene expression analysis was conducted to verify the changes in gene expression in oxaliplatin‐resistant RKO cells using RNA sequencing (RNA‐seq). This aimed to compare the gene expression of RKO cells cultured in normal medium with that of RKO cells cultured in a medium with 50% concentration of bacterial culture filtrate. The RNA‐seq results are depicted as a volcano plot in Figure 2A. Since the data included pseudogenes, these were excluded from the analysis to focus on functional protein‐coding genes. As a result, eight significantly downregulated genes were selected for further investigation. These eight genes were targeted for knockdown via siRNA transfection into RKO cells, and resistance to oxaliplatin was then assessed using an MTT assay (Figure 2B). The control showed a mean cell viability of 29.1% ± 4.2% (SD), whereas the *Ninjurin2* (*NINJ2*) knockdown cells exhibited a higher cell viability of 41.6% ± 4.3%. Among the genes tested, only *NINJ2* knockdown resulted in a significant increase in cell viability in the MTT assay (*p* = 0.043). To further evaluate this resistance, two other *NINJ2* gene siRNAs (*NINJ2 si#2* and *NINJ2 si#3*) were transfected into RKO cells (Figure 2C). Results demonstrated that cell viability was elevated in RKO cells transfected with any of the three *NINJ2* gene siRNAs when compared to those with the control siRNA. Specifically, cell viability was 41.3% ± 1.7% for control siRNA, 52.5% ± 4.9% for *NINJ2* si#1 (*p* = 0.039), 48.5% ± 2.6% for *NINJ2* si#2 (*p* = 0.028), and 52.2% ± 3.4% for *NINJ2* si#3 (*p* = 0.016). *NINJ2* mRNA levels were analyzed using qPCR as shown in Figure 2D. When the control was set to 1, the results showed that *NINJ2* si#1 had a mean level of 2.1 × 10^−5^ ± 0.5 × 10^−5^, *NINJ2* si#2 had a mean level of 2.0 × 10^−4^ ± 0.2 × 10^−4^, and *NINJ2* si#3 had a mean level of 1.3 × 10^−5^ ± 0.2 × 10^−5^, indicating that the *NINJ2* gene was effectively suppressed in each case. These findings suggest that the *NINJ2* gene plays a pivotal role in the oxaliplatin resistance induced by bacterial culture filtrates. Furthermore, the *NINJ2* expression levels in RKO, DLD1, HT29, and HCT116 were measured using qPCR (Figure 2E). When RKO was set to 1, the average expression levels were 0.68 ± 0.13 for DLD1, 2.8 × 10^−3^ ± 0.6 × 10^−3^ for HT29, and 3.0 × 10^−3^ ± 1.6 × 10^−3^ for CO115. RKO and DLD1 exhibited higher *NINJ2* expression levels compared to HT29 and CO115. *NINJ2* expression levels were higher in RKO compared to HT29 (*p* < 0.01) and HCT116 (*p* < 0.01).

### 3.3 Relation of *NINJ2* and Resistance of RKO Cell to Oxaliplatin

**FIGURE 3:**
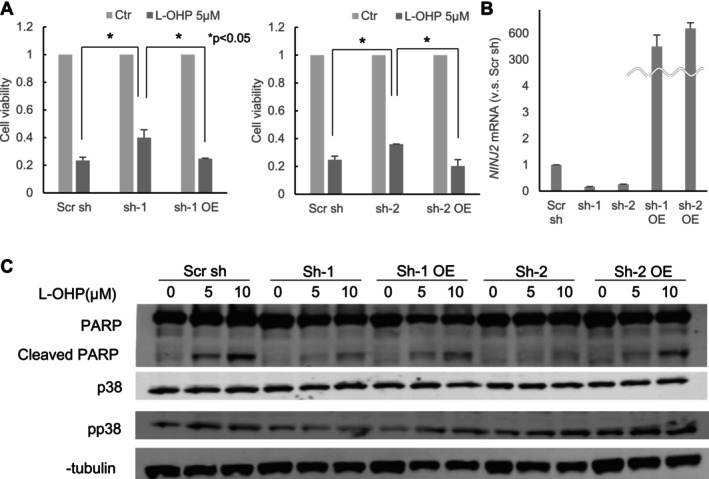
Examination of the correlation between *NINJ2* gene expression and oxaliplatin resistance. (A) Cells of Scr sh, sh‐1, sh‐1 OE, sh‐2, and sh‐2 OE were seeded and incubated for 24 h, then treated at a final concentration of 5 μM oxaliplatin (L‐OHP). An MTT assay was conducted 48 h post‐oxaliplatin treatment. The ratio of (cell viability post‐oxaliplatin treatment) to (cell viability post‐saline treatment) is depicted on the vertical axis *p < 0.05. (B) RNA was extracted from Scr sh, sh‐1, sh‐2, sh‐1 OE, and sh‐3 OE cells, followed by RT‐PCR analysis. The ratios of (mRNA in sh‐1 cells to mRNA in Scr cells), (mRNA in sh‐2 cells to mRNA in Scr cells), (mRNA in sh‐1 OE cells to mRNA in Scr cells), and (mRNA in sh‐2 OE cells to mRNA in Scr cells) are illustrated on the vertical axis. (C) Scr sh, sh‐1, sh‐2, sh‐1 OE, and sh‐2 OE cells were treated with saline or with oxaliplatin. Protein extraction was performed on the harvested cells followed by Western blot analysis.

ShRNA targeting the *NINJ2* gene was transfected into RKO cells to establish stable *NINJ2* gene knockdown cell lines (sh‐1, sh‐2). The resistance of sh‐1 and sh‐2 cell lines to oxaliplatin was examined using the MTT assay (Figure 3A). The mean cell viability of RKO cells transfected with control scrambled shRNA (Scr sh) was 23.4% ± 3.3%, compared to 40.0% ± 8.1% in sh‐1 cells (*p* = 0.028). Similarly, the viability of sh‐2 cells had a mean of 35.9% ± 0.5%, while the Scr sh control was 24.7% ± 3.7% (*p* = 0.013). Both sh‐1 and sh‐2 exhibited higher resistance to oxaliplatin than Scr sh. The expression level of the *NINJ2* gene in sh‐1 and sh‐2 was validated by qPCR (Figure 3B). When the *NINJ2* expression level of Scr sh was set to 1, sh‐1 had a mean expression level of 0.16 ± 0.02 (*p* < 0.01), and sh‐2 had a mean of 0.26 ± 0.02 (*p* < 0.01). These results confirm the suppression of *NINJ2*. A lentiviral plasmid vector expressing the *NINJ2* gene was subsequently infected into the sh‐1 and sh‐2 cell lines to overexpress the *NINJ2* gene. This led to the establishment of *NINJ2* overexpression cell lines based on sh‐1 and sh‐2 (sh‐1 OE, sh‐2 OE). The upregulation of the *NINJ2* gene was verified by qPCR (Figure 3B). When the *NINJ2* expression level of Scr sh was set to 1, the mean expression level of sh‐1OE was 342 ± 129 (*p* < 0.01), and that of sh‐2OE was 619 ± 67 (*p* < 0.01). The resistance of sh‐1 and sh‐2 to oxaliplatin was again assessed using the MTT assay (Figure 3A). The mean cell viability of sh‐1 OE was 26.1% ± 1.7%, and that of sh‐2 OE was 20.2% ± 6.5%. Both sh‐1 OE and sh‐2 OE exhibited lower resistance to oxaliplatin compared to sh‐1 and sh‐2 (sh‐1 OE, *p* = 0.029; sh‐2OE, *p* = 0.028). These results indicate that the overexpression of *NINJ2* reversed oxaliplatin resistance.

To discern the oxaliplatin‐induced alterations in protein expression across sh‐1, sh‐2, sh‐1 OE, and sh‐2 OE cell lines, a Western blot analysis was carried out (Figure 3C). In the cell line transfected with Scr sh, apoptosis‐related proteins, including cleaved PARP and pp38, were induced in an oxaliplatin concentration‐dependent manner. However, in the *NINJ2* knockdown cell lines (sh‐1, sh‐2), this protein upregulation following oxaliplatin treatment was suppressed. Yet, in the *NINJ2* overexpression cell lines (sh‐1 OE, sh‐2 OE), the proteins cleaved PARP and pp38 were induced in an oxaliplatin concentration‐dependent manner, mirroring the cell line transfected with Scr sh. Such findings suggest that the suppression of apoptosis induction may be one mechanism underlying oxaliplatin resistance when RKO cells were treated with bacterial culture filtrate.

### 3.4 Inhibition of Apoptosis Induction by *NINJ2* Gene Suppression

**FIGURE 4:**
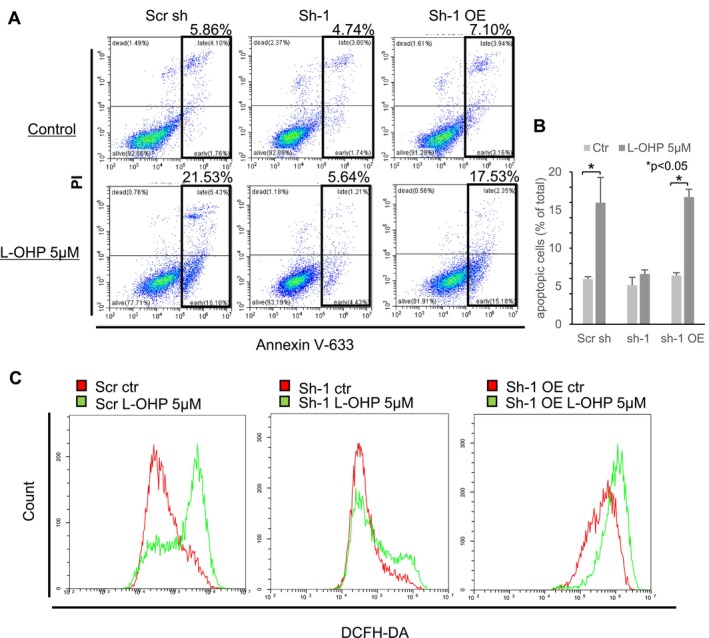
Exploration of the association between *NINJ2* gene expression and apoptosis induced by oxaliplatin. (A) Cells labeled as Scr sh, sh‐1, and sh‐1 OE were treated with oxaliplatin (L‐OHP) at a final concentration of 5 μM. Forty‐eight hours after treatment, the number of apoptosis‐induced cells was determined using flow cytometry. The percentages shown are from a representative single experiment. (B) The bar graph represents the percentage of apoptotic cells. The percentages shown are the average of three independent experiments *p < 0.05. (C) Cells designated as Scr sh, sh‐1, and sh‐1 OE were treated with oxaliplatin at a final concentration of 5 μM. ROS‐positive cells were measured 48 h after oxaliplatin treatment using the Highly Sensitive DCFH‐DA ROS Assay Kit.

To further validate that the suppression of *NINJ2* gene expression attenuates oxaliplatin‐induced apoptosis, annexin V assays were conducted (Figure 4A). In the cell line transfected with Scr sh, the mean proportion of apoptotic cells increased from 5.9% ± 0.4% to 16.0% ± 4.7% after oxaliplatin treatment (Figure 4B, *p* = 0.039). In contrast, the mean proportion of apoptotic cells following oxaliplatin treatment increased slightly in the cell line with reduced *NINJ2* gene expression (sh‐1), changing from 5.1% ± 1.5% to 6.6% ± 0.7%. This proportion of apoptotic cells rose again in the *NINJ2*‐overexpressed cell line (sh‐1 OE), changing from 6.4% ± 0.5% to 16.7% ± 1.4% (*p* < 0.01). Mirroring the results of the Western blot analysis, this annexin V assay indicated that the inhibition of apoptosis induction is central to the resistance of *NINJ2* knockdown cells to oxaliplatin.

Given that oxaliplatin induces DNA damage and apoptosis by forming cross‐links in DNA and generating ROS [41, 42], flow cytometry analysis was executed to assess ROS production in control, sh‐1, and sh‐1 OE cell lines post‐oxaliplatin treatment (Figure 4C). Oxaliplatin treatment amplified the fluorescence signal in the control (Scr sh) and *NINJ2*‐overexpressed cells (sh‐1 OE). Conversely, there was no notable impact on the fluorescence signal in the *NINJ2*‐suppressed cells (sh‐1). This data imply that *NINJ2* gene suppression could inhibit the production of oxaliplatin‐induced ROS.

### 3.5 Validation of the Relationship Between *NINJ2* Gene Expression and Oxaliplatin Resistance in the Xenograft Mouse Model

**FIGURE 5:**
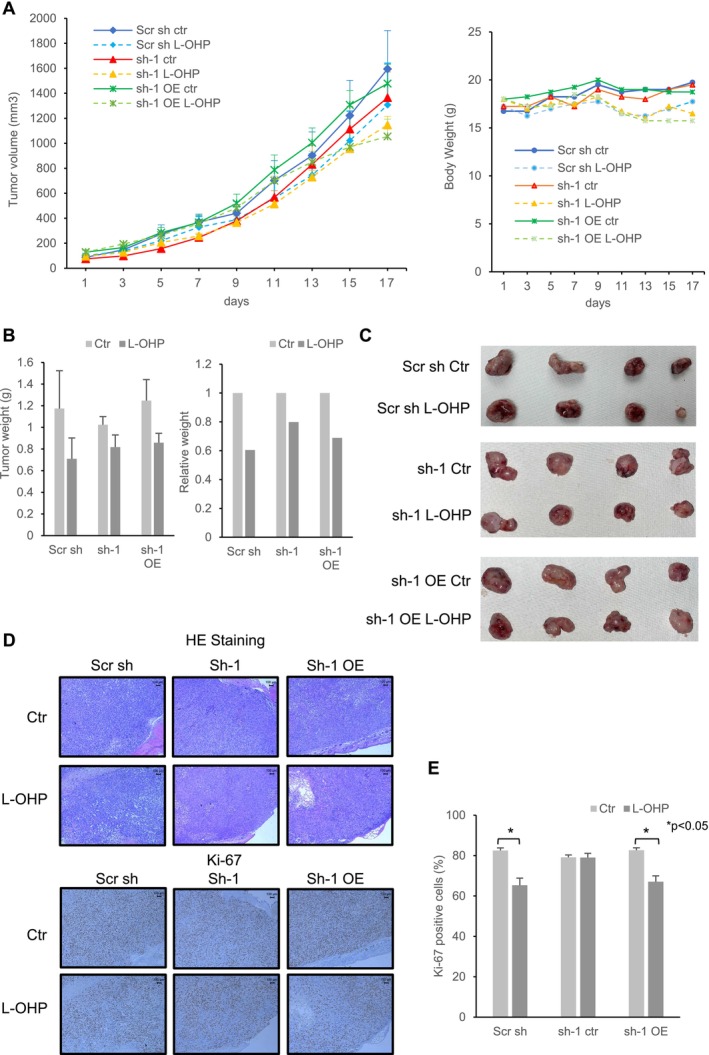
Examining the impact of *NINJ2* expression on oxaliplatin's antitumor effect in a tumor xenograft mouse model. (A) Tumor cells (1.0 × 10^7^ cells) were subcutaneously transplanted into the lateral abdomen of 4‐week‐old nude mice (female, BALB/c‐nu). Mice were then randomly allocated into the Scr sh group, sh‐1 group, and sh‐1 OE group. Four mice from each group received culture medium treatment, while the remaining four from each group were administered 10 mg/kg oxaliplatin intraperitoneally. Line graphs depict the variations in tumor volume and body weight across the groups during the observation phase. Tumor volume was calculated using the formula: Tumor volume (mm^3^) = 0.5 × length (mm) × width (mm)^2^. (B) On day 18 post‐initiation of oxaliplatin treatment, the average tumor weights from each group are provided. The vertical axis represents the ratio of the average tumor weight from oxaliplatin‐treated mice to that of the culture medium‐treated mice. (C) Representative image of tumors extracted from each group on the 18th day after commencing oxaliplatin treatment are displayed. (D) Both HE staining and immunohistochemical staining utilizing Ki‐67 antibody were conducted on tumors from each group (100× magnification). (E) The average proportion of Ki‐67 positive cells in each group is shown. The vertical axis represents the proportion of Ki‐67 positive cells *p < 0.05.

To assess the association between *NINJ2* gene expression and oxaliplatin resistance, an in vivo study was conducted (Figure 5A–C). On the 18th day after the injection of scrambled shRNA cells (Scr sh), the mean tumor volume in control mice was 1590 mm^3^ ± 532 mm^3^, whereas it decreased to 1310 mm^3^ ± 333 mm^3^ in mice treated with oxaliplatin (Figure 5A). However, this difference was not statistically significant. This trend was also observed in sh‐1 and sh‐1 OE cells. Similarly, the body weight of the mice on day 18 after the injection of Scr sh cells was 19.75 g ± 2.57 g in control mice, compared to a lower average of 17.75 g ± 2.96 g in those treated with oxaliplatin. This difference was also not statistically significant, and a similar trend was observed in sh‐1 and sh‐1 OE cells. In terms of tumor weight (Figure 5B), mice injected with Scr sh cells showed a reduction from a mean of 1.17 g ± 0.61 g to 0.71 g ± 0.34 g following oxaliplatin treatment. In mice injected with sh‐1 cells, the tumor weight slightly decreased from a mean of 1.02 g ± 0.13 g to 0.82 g ± 0.19 g, while in mice injected with sh‐1 OE cells, the tumor weight decreased from 1.25 g ± 0.34 g to 0.86 g ± 0.15 g. However, the differences were not statistically significant. When comparing the relative tumor weight, the ratios were 60% in Scr sh cells and 68% in sh‐1 OE cells, compared to 80% in sh‐1 cells. This suggests a trend where *NINJ2* downregulation may attenuate the antitumor effect of oxaliplatin in vivo.

To measure changes in tumor growth potential due to the modulation of *NINJ2* gene expression, immunohistochemical staining with the Ki‐67 antibody was performed (Figure 5D). In the *NINJ2*‐downregulated tumors (sh‐1), the mean proportion of Ki‐67 positive cells remained unchanged before and after oxaliplatin treatment (Figure 5E). In contrast, in Scr sh tumors, the proportion significantly decreased from 82.6% ± 1.8% to 65.3% ± 5.0% (*p* = 0.010), and in sh‐1 OE tumors, it decreased significantly from 82.7% ± 1.7% to 67.1% ± 4.1% following oxaliplatin treatment (*p* < 0.01).

## 4 Discussion

In this study, introducing bacterial culture filtrate to the culture medium of colon cancer cells induced resistance to oxaliplatin. An underlying mechanism identified was the downregulation of the *NINJ2* gene expression by the bacterial culture filtrate, which subsequently contributed to oxaliplatin resistance in colon cancer cells.

The MTT assay indicated that the resistance of cancer cells to oxaliplatin, induced by the addition of bacterial culture filtrate, is cell line dependent. These results suggest that the bacterial culture filtrate modifies the colon cancer cells, making them resistant to oxaliplatin. Importantly, this alteration does not seem to result from the direct degradation of oxaliplatin by the bacterial culture filtrate.

There are various types of EVs, including not only outer membrane vesicles secreted by gram‐negative bacteria, but also microvesicles (100–1000 nm) and apoptotic bodies (1000–5000 nm) [43], suggesting that a significant number of EVs would be excluded by a 0.2 μm filter. EVs derived from gram‐negative bacteria have been reported to range in size from 20 to 250 nm [37, 44], although their size can vary depending on the bacterial species and may be altered by antibiotic treatment [45, 46]. Therefore, the vesicles that are most likely to affect oxaliplatin resistance in this study can pass through the filter. EVs are considered a potential factor contributing to the resistance of colon cancer cells to oxaliplatin. These vesicles, known to be secreted by bacteria, carry RNA, DNA, and proteins [32, 37]. Furthermore, EVs have been implicated in promoting tumor growth and metastasis due to the molecular contents they transport [35, 36]. In the context of this study, it is plausible that EVs secreted by *Citrobacter freundii* induced resistance in CRC cells to oxaliplatin. This could be achieved by downregulating *NINJ2* gene expression via specific molecules housed within the EVs. Nevertheless, the exact molecules and mechanisms at play remain unidentified. Instead, this study focused on the gene expression changes induced by the bacterial culture filtrate. While isolating and analyzing EVs demands intricate techniques, advancements like size exclusion chromatography and microfluidic device methods have been developed for isolation [47, 48]. In fact, there has been a report demonstrating that EVs secreted by gut bacteria are involved in colorectal cancer development [49]. Future studies will focus on investigating the mechanism by which EVs in bacterial culture filtrates contribute to oxaliplatin resistance. Another objective is to compare the EVs found in bacterial filtrates with those present in the intestines in vivo.

The *NINJ2* gene is located on chromosome 12p13 and encodes a cell adhesion factor [50]. Mutations in the *NINJ2* gene have been linked to an increased risk of stroke and multiple sclerosis [51, 52]. Additionally, elevated *NINJ2* gene expression has been observed in fibrotic liver cells [53]. However, the precise function of the *NINJ2* gene remains elusive. Knocking down the *NINJ2* gene in vascular endothelial cells suppresses inflammatory cytokines such as IL‐1β through TLR4 [54]. IL‐1β, a potent inflammatory cytokine known to induce tissue damage, is associated with the generation of intracellular ROS [55, 56]. In our study, we established that downregulation of the *NINJ2* gene reduced ROS production during oxaliplatin treatment. Given that oxaliplatin has been reported to stimulate intracellular ROS production [41, 42], the inhibition of ROS generation through *NINJ2* gene downregulation might elucidate the resistance to oxaliplatin seen in colon cancer cells. Conversely, 5‐FU, which is known to induce minimal ROS generation [41], did not lead to oxaliplatin resistance in colon cancer cells, even when supplemented with bacterial culture filtrate (Figure 1B). This minimal ROS generation by 5‐FU might explain the absence of resistance observed in colon cancer cells, even with *NINJ2* gene downregulation. Among platinum drugs known for robust ROS generation, only oxaliplatin displayed resistance in our study (Figure 1B). Platinum drugs are complexes comprised of platinum with a desorbing group and a carrier group. The desorbing group detaches to form the active, cytotoxic entity [57]. While cisplatin and carboplatin share a common carrier group and transition into the same active form within the cell, oxaliplatin boasts a unique carrier group, leading to distinct pharmacological effects. This structural variation between oxaliplatin and its platinum counterparts is believed to induce not just DNA damage but also intracellular protein damage [42]. This differential effect might underpin the observed variances in drug resistance in our study.

The resistance to oxaliplatin induced by bacterial culture filtrate varied across the different cell lines. Mutations in key oncogenes, tumor suppressor genes, and the MSI status of the CRC cell lines used in this study were compared based on a previous report [58]. However, no mutation patterns could explain the differences in oxaliplatin resistance induced by the bacterial filtrate (Table S1). In contrast, *NINJ2* expression levels of RKO and DLD1 were higher than those of HT29 and HCT116 (Figure 2E). This suggests that *NINJ2* expression levels may influence oxaliplatin resistance.

In the xenograft mouse model, tumors with downregulated *NINJ2* expression displayed a tendency to resist shrinkage upon oxaliplatin treatment (Figure 5B). Moreover, the reduction in Ki‐67‐positive cells typically caused by oxaliplatin was mitigated by the downregulation of the *NINJ2* gene (Figure 5E). These observations suggest that *NINJ2* gene downregulation also diminishes the antitumor effect of oxaliplatin in vivo. However, due to the rapid proliferation and variability in tumor size of RKO cells, statistically significant differences could not be obtained (Figure 5A,B). Experiments using other cell lines are under consideration. While *this* in vivo study focused on evaluating oxaliplatin resistance associated with *NINJ2* expression, future work will involve administering *Citrobacter freundii* directly into the mouse intestine to further investigate its role in oxaliplatin resistance. Additionally, there have been no reports investigating *NINJ2* expression levels in CRC patients. Investigating the relationship between *NINJ2* expression and oxaliplatin resistance in clinical trials with CRC patients will also be an important future direction.

To the best of our knowledge, this is the first report demonstrating that the inclusion of bacterial culture filtrate leads to genetic alterations, and that the downregulation of the *NINJ2* gene correlates with resistance to anticancer drugs. *NINJ2* expression might serve as a predictive biomarker for the therapeutic efficacy of oxaliplatin. Manipulating *NINJ2* gene expression could pave the way for innovative therapeutic approaches to enhance oxaliplatin treatment outcomes.

## Author Contributions

**Reio Ueta:** data curation (equal), formal analysis (equal), investigation (equal), methodology (equal), validation (equal), visualization (equal), writing – original draft (equal), writing – review and editing (equal). **Hiroo Imai:** conceptualization (equal), data curation (equal), formal analysis (equal), funding acquisition (equal), investigation (equal), methodology (equal), project administration (equal), validation (equal), visualization (equal), writing – original draft (equal), writing – review and editing (equal). **Ken Saijo:** formal analysis (equal), methodology (equal), project administration (equal), supervision (equal), validation (equal), writing – review and editing (equal). **Yoshifumi Kawamura:** data curation (supporting), formal analysis (equal), methodology (supporting), validation (supporting). **Shuto Kodera:** data curation (supporting), formal analysis (supporting), validation (equal). **Chikashi Ishioka:** formal analysis (equal), methodology (equal), project administration (equal), supervision (equal), writing – review and editing (equal).

## Ethics Statement

All animal experiments adhered to institutional guidelines set by Tohoku University and received approval from the Institutional Animal Care and Use Committee of Tohoku University (approval number. 2023 MdA‐059).

## Conflicts of Interest

Chikashi Ishioka has received research funding from the Tokyo Cooperative Oncology Group. Furthermore, contributions have been accepted from Chugai Pharmaceutical; Novartis Pharma K.K.; Ono Pharmaceutical; MSD; Pfizer; AstraZeneca; Bristol‐Myers Squibb; Kyowa Kirin Co. Ltd.; Janssen Pharmaceutical; Taiho Pharmaceutical; Daiichi Sankyo Company, Limited; Takeda Pharmaceutical; Merck Biopharma Co. Ltd.; Eli Lilly Japan K.K.; Bayer Yakuhin Ltd.; and Incyte Biosciences Japan G.K. Chikashi Ishioka is a representative of the Tohoku Clinical Oncology Research and Education Society, a nonprofit organization. The authors report no other conflicts of interest in this work.

## Supporting information


**Figure S1:** Assessment of oxaliplatin sensitivity influenced by *Pseudomonas alcaliphila* bacterial culture filtrate. RPMI medium and *Pseudomonas alcaliphila* bacterial culture filtrate were combined at specified concentrations (0%, 10%, 20%, 30%, 40%, and 50%). Cells were then incubated and treated with oxaliplatin (L‐OHP) at 5 μM concentration following a 24‐h period. An MTT assay was carried out 48 h post‐oxaliplatin treatment. The resulting absorbance and cell viability are represented on the vertical axis, with cell viability displayed relative to its level at each bacterial culture filtrate concentration *p < 0.05.

**Table S1:** Colon cancer cell lines classified by key gene mutations and MSI status. MSI, microsatellite instability; MSS, microsatellite stable; WT, wild type. Data from Ahmed D, et al. (2013). (58).

## Data Availability

The data that support the findings of this study are available from the corresponding author upon reasonable request.
