# Supplementary figures and images for: Influence of Citrobacter freundii on NINJ2 Expression and Oxaliplatin Resistance in Colorectal Cancer
Source: Cancer Med. 2025 Jun 26;14(13):e70940. doi: 10.1002/cam4.70940 (PMC12198655; doi:10.1002/cam4.70940)

## Slide 1
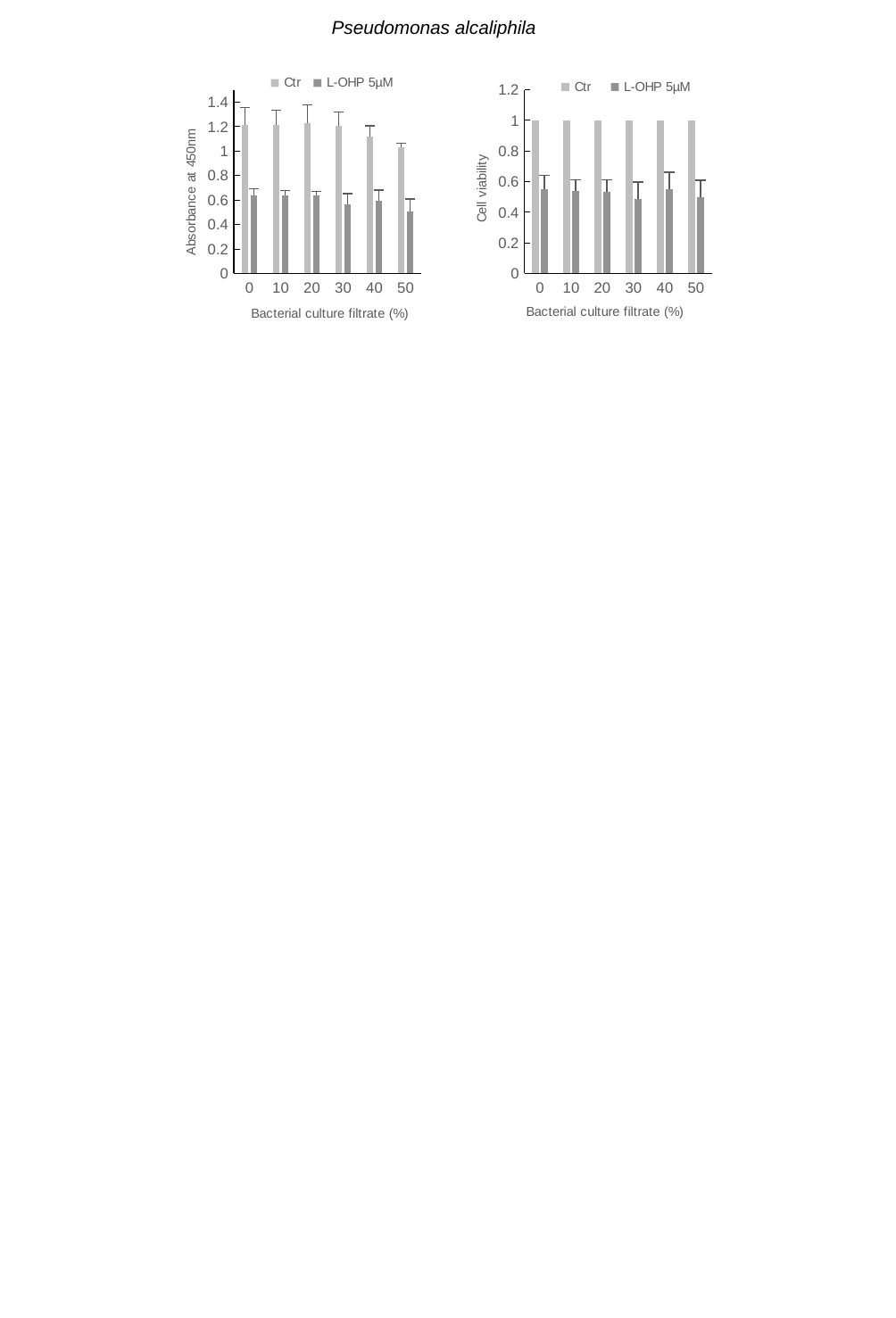

Pseudomonas alcaliphila
### Chart
| Category | Ctr | L-OHP 5µM |
|---|---|---|
| 0 | 1.2152999999999998 | 0.6411333333333333 |
| 10 | 1.2147666666666668 | 0.6377 |
| 20 | 1.2282666666666666 | 0.6341 |
| 30 | 1.2058 | 0.5624666666666667 |
| 40 | 1.1167 | 0.5970666666666666 |
| 50 | 1.0278666666666665 | 0.5054333333333333 |
### Chart
| Category | Ctr | L-OHP 5µM |
|---|---|---|
| 0 | 1.0 | 0.5487215123203436 |
| 10 | 1.0 | 0.5387008789169005 |
| 20 | 1.0 | 0.5332465939592569 |
| 30 | 1.0 | 0.4870984517282917 |
| 40 | 1.0 | 0.5523471107492205 |
| 50 | 1.0 | 0.4980851203335881 |

Supplement: Supplementary file 1 — Figure S1: Assessment of oxaliplatin sensitivity influenced by Pseudomonas alcaliphila bacterial culture filtrate. RPMI medium and Pseudomonas alcaliphila bacterial culture filtrate were combined at specified concentrations (0%, 10%, 20%, 30%, 40%, and 50%). Cells were then incubated and treated with oxaliplatin (L‐OHP) at 5 μM concentration following a 24‐h period. An MTT assay was carried out 48 h post‐oxaliplatin treatment. The resulting absorbance and cell viability are represented on the vertical axis, with cell viability displayed relative to its level at each bacterial culture filtrate concentration *p < 0.05. [file CAM4-14-e70940-s001.pptx]
