# Supplementary material for: Influence of Citrobacter freundii on NINJ2 Expression and Oxaliplatin Resistance in Colorectal Cancer
Source: Cancer Med. 2025 Jun 26;14(13):e70940. doi: 10.1002/cam4.70940 (PMC12198655; doi:10.1002/cam4.70940)
Supplement: Supplementary file 2 — Table S1: Colon cancer cell lines classified by key gene mutations and MSI status. MSI, microsatellite instability; MSS, microsatellite stable; WT, wild type. Data from Ahmed D, et al. (2013). (58). [file CAM4-14-e70940-s002.docx]

Supplementary table 1. Colon cancer cell lines classified by key gene mutations and MSI status

| **Cell line** | **BRAF** | **KRAS** | **PIK3CA** | **PTEN** | **TP53** | **MSI status** |
| --- | --- | --- | --- | --- | --- | --- |
| **RKO** | V600E | WT | H1047R | WT | WT | MSI |
| **DLD1** | WT | G13D | E545K; D549N | WT | S241F | MSI |
| **HT29** | V600E | WT | P449T | WT | R273H | MSS |
| **HCT116** | WT | G13D | H1047R | WT | WT | MSI |

Abbreviations: MSI, microsatellite instability; MSS, microsatellite stable; WT, wild type

Data from *Ahmed D, et al. (2013)*. (58)
